# Comprehensive data resources and analytical tools for pathological association of aminoacyl tRNA synthetases with cancer

**DOI:** 10.1093/database/bav022

**Published:** 2015-03-29

**Authors:** Ji-Hyun Lee, Sungyong You, Do Young Hyeon, Byeongsoo Kang, Hyerim Kim, Kyoung Mii Park, Byungwoo Han, Daehee Hwang, Sunghoon Kim

**Affiliations:** ^1^Medicinal Bioconvergence Research Center and ^2^Research Institute of Pharmaceutical Sciences, College of Pharmacy, Seoul National University, Seoul 151-742, Republic of Korea, ^3^School of Interdisciplinary Bioscience and Bioengineering, POSTECH, Pohang 790-784, Republic of Korea, ^4^Department of New Biology and Center for Plant Aging Research, Institute for Basic Science, DGIST, Daegu 711-873, Republic of Korea and ^5^Department of Molecular Medicine and Biopharmaceutical Sciences, Seoul National University, Seoul 151-742, Republic of Korea

## Abstract

Mammalian cells have cytoplasmic and mitochondrial aminoacyl-tRNA synthetases (ARSs) that catalyze aminoacylation of tRNAs during protein synthesis. Despite their housekeeping functions in protein synthesis, recently, ARSs and ARS-interacting multifunctional proteins (AIMPs) have been shown to play important roles in disease pathogenesis through their interactions with disease-related molecules. However, there are lacks of data resources and analytical tools that can be used to examine disease associations of ARS/AIMPs. Here, we developed an Integrated Database for ARSs (IDA), a resource database including cancer genomic/proteomic and interaction data of ARS/AIMPs. IDA includes mRNA expression, somatic mutation, copy number variation and phosphorylation data of ARS/AIMPs and their interacting proteins in various cancers. IDA further includes an array of analytical tools for exploration of disease association of ARS/AIMPs, identification of disease-associated ARS/AIMP interactors and reconstruction of ARS-dependent disease-perturbed network models. Therefore, IDA provides both comprehensive data resources and analytical tools for understanding potential roles of ARS/AIMPs in cancers.

**Database URL:** http://ida.biocon.re.kr/, http://ars.biocon.re.kr/

## Introduction

Cells have an array of cytoplasmic and mitochondrial aminoacyl-tRNA synthetases (ARSs) that are involved in cellular protein synthesis. ARSs catalyze the ligation of amino acids to their cognate tRNAs during protein synthesis. Thus, ARSs have been considered as housekeepers involved in protein synthesis, being less sensitive to system perturbation compared with signal mediators or transcriptional factors that are fully dedicated to system control. However, recently, aberrant expression, mislocalization and variant formation of ARSs have been observed in various cancer cells (1). For this reason, more attention is being paid to potential roles of ARS/ARS-interacting multifunctional proteins (AIMPs) in system regulation and pathogenesis of various diseases.

Mammalian ARSs contain additional domains attached to their catalytic domains, compared with prokaryotic counterparts (2). Using these additional domains, they interact with other molecules to form diverse complexes, thereby affecting activities of disease-related cellular processes (3). More intriguingly, several mammalian ARSs form a macromolecular protein complex with three non-enzymatic factors named AIMPs (4, 5). Although they are not the enzymes like ARSs, they are considered as the members of the ARS community since they play critical scaffolding role in the structural integrity of the multi-tRNA synthetase complex (MSC). Thus, we consider three AIMPs together with 20 cytosolic ARSs as the same community group. A growing volume of evidence shows that ARS/AIMPs are closely associated with disease pathogenesis through their interactions with disease-related molecules (1, 6). These data indicate that alterations in interactions of ARS/AIMPs, together with abnormal expression and mislocalization of ARS/AIMPs, can lead to perturbation of disease-related cellular networks.

Huge amounts of genomic, transcriptomic, proteomic and interaction data for diverse diseases have been accumulated. Recently, the importance of ARS/AIMPs in cancer pathogenesis has been addressed in a series of publications (7–14). Thus, we previously reviewed potential roles of ARS/AIMPs in various cancers by reanalyzing previously published global datasets (1). However, exploration of abnormal expression and interaction of ARS/AIMPs in these diseases is limited due to the lack of comprehensive resources that can be used to effectively navigate and explore alterations in genomic, transcriptomic and proteomic data and also in interactions of ARS/AIMPs in various diseases. Furthermore, there is a severe lack of analytical tools that can be used to analyze associations of ARS/AIMPs with human diseases and to reconstruct ARS/AIMP-dependent disease-perturbed network models. Here, we present an Integrated Database for ARS/AIMPs (IDA) that provides (i) genomic, transcriptomic and proteomic data [mRNA expression, somatic mutation, copy number variation (CNV) and phosphorylation data] of ARS/AIMPs and their interactors in various cancers and (ii) protein–protein interactions (PPIs) of ARS/AIMPs.

## Materials and methods

### Database and website infrastructure

IDA is based on the relational database management system for data storage and integration with external data sources. Gene expression and protein-interaction data are stored in a MySQL database. Identifiers of all probes on the microarray types for gene expression data, Ensembl and dbSNP identifiers for genomic mutation data, identifiers of international protein index or UniProtKB/Swiss-Prot for protein expression or post-translational modifications (PTMs) and all identifiers for PPIs in the interactome databases have been converted into NCBI Entrez identifiers. Using the relationships among different identifiers, we have optimized the database schema (Supplementary Figure S1) to improve the response time of the database when gene/protein expression, genomic mutation, PTM and PPI data are searched for. Moreover, IDA provides a web-based user interface for data exploration and visualization. Cancer-associated differential expression, mutation and CNVs of ARS/AIMPs and their first and second neighbors were summarized using several visualization tools, such as two-way heat maps, a pinwheel-shaped heat map and bar graphs, respectively. These data for individual genes can be visualized using profile plots or extracted in a tubular format. Finally, two different network analysis tools, Cancer-associated network and Functional modules are servicing. IDA web site is constructed with PHP 5.3.3, SWFObject v1.5, MATLAB R2011a, MySQL 5.1.71, CentOS release 6.5 and Apache/2.2.15 (Unix).

### Identification of differentially expressed genes

IDA includes 40 cancer gene expression datasets. For the 36 cancer datasets generated from Affymetrix microarray platforms, the log_2_ intensities in each gene expression dataset were normalized using the GC-RMA normalization method (15). For the other four datasets generated from Illumina, ABI and custom microarray platforms, the log_2_-intensities were normalized using the quantile normalization method (16). For each dataset, the expressed genes were then identified using a Gaussian mixture model method as previously described (17). Briefly, in the mixture modeling, the distribution of the observed probe intensity (probeset intensity for Affymetrix data) was fitted by two Gaussian probability density functions: one was for expressed genes while the other was for non-expressed genes. We then selected the expressed genes as the ones whose maximum intensities across all samples in the dataset were higher than the threshold intensity at which the two fitted Gaussian probability density functions meet. To identify differentially expressed genes (DEGs) between cancer and normal samples, an integrative statistical hypothesis testing was performed (18, 19). Briefly, Student *t*-test and log_2_-median-ratio test were applied to calculate *T* values and log_2_-median-ratios for all the genes. Empirical distributions of the null hypothesis were then estimated by performing random permutations of samples and then by performing the kernel density estimation (20) to *T* values and log_2_-median-ratios resulted from the random permutations. Next, the adjusted *P* values of each gene for the individual tests were computed by the two-tailed test using the empirical distribution. The *P* values from the two tests were then combined using Stouffer’s method (19). The DEGs were identified as the genes with *P* ≤ 0.05. Finally, as the representative *P* values of the genes in multiple datasets of each cancer type, the combined *P* values of genes in individual datasets were summarized using Stouffer’s method (19).

### Deregulation and co-association scores for nodes and edges in the network models

For the nodes (ARS/AIMPs and there first cancer-associated neighbors) and edges in the ARS/AIMP cancer-associated network, the two types of cancer association scores model were estimated. First, the deregulation score for each node which denotes the representative degree of deregulation in the 10 cancer types for which gene expression data were collected. The combined *P* values of each node in 10 different cancer types were first calculated. These *P* values were then transformed to Z values using the inverse standard normal cumulative distribution. The cumulative sum of Z values of its direct interactors for each node was calculated in each cancer type (21). Finally, the average of the cumulative sum of *Z* values in 10 cancer types was computed as the deregulation score. Second, the co-association score between an ARS/AIMP and their first neighbors representing the extent of co-deregulation in 10 cancer types is estimated as ‘the number of cancer types where the interacting pairs of ARS/AIMP-first neighbors are commonly differentially expressed’ divided by ‘the total number of cancer types’.

### Identification of key modules in the ARS/AIMP network model

Key modules in the ARS/AIMP network were identified using the random walk with restart (RWR) algorithm previously reported (22). Briefly, in the RWR algorithm, we used all nodes in the network as the seeds and assigned equal initial probabilities (*p^t^* at *t* = 0) to the seeds. Random walks then started with the transitions rate of probability = 0.75 and then stopped until L_1_ norm of the difference between *P^t^* and *P^t+^^1^* (random walk probabilities of the nodes at *t* and *t*+1, respectively) became <10^−6^. To evaluate the significance of *p^t^*, we repeated the random walk process for randomized networks where the edges were randomly permuted, estimated an empirical null distribution of *P^t^* resulted from the randomized networks and *P* values of *P^t^* for the nodes in the real network by the right-sided test using the empirical null distribution. From the ARS/AIMP network, we finally identified a set of subnetworks including the nodes with *P* < 0.05 and defined the subnetworks as key modules.

### Identification of evolutionarily conserved motifs in the ARS/AIMP network model

To identify evolutionarily conserved regulatory motifs, we first mapped the nodes in the ARS/AIMP network model for each species into the ortholog groups in eggnog (23) and then removed redundant interactions and self-interactions arising from many-to-one mapping. In the network model for each species, regulatory motifs were searched using the ortholog groups, and the overlapped ones among the species were identified as evolutionarily conserved ones. The interactions among the ortholog groups in the conserved motifs were then converted to all the possible protein interactions. After the conversion, only the motifs composed of the interactions existing in the real interactome were selected. Finally, among them, the ones including at least one of ARS/AIMPs were selected as a final list of evolutionarily conserved motifs.

## Results and discussion

### Comprehensive data resources of ARS/AIMPs and their interactors in IDA

#### Cancer-associated genes

**Figure 1.**
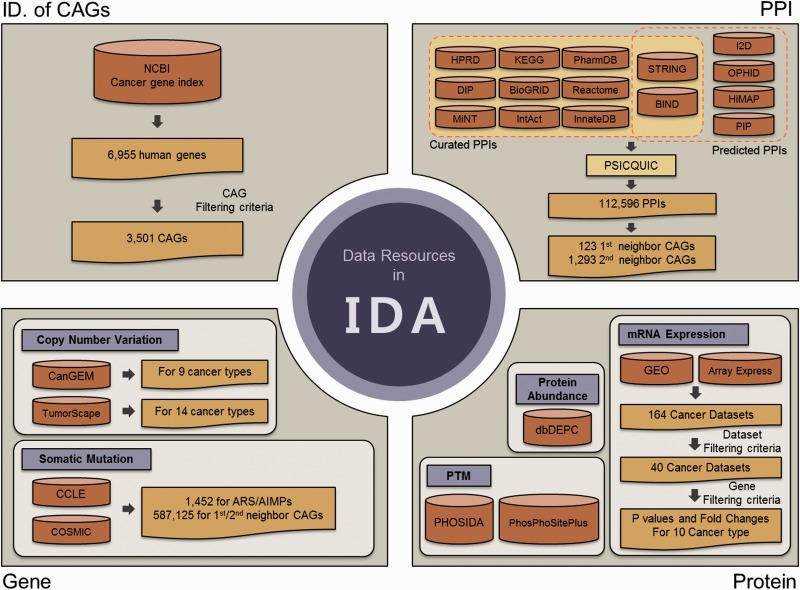
Data resources in IDA. IDA contains CAGs and genomic, transcriptomic, proteomic and PPIs for ARS/AIMPs and their first and second neighbor CAGs. First, IDA provides 3501 CAGs selected from the 6955 genes in NCBI cancer gene index based on the filtering criteria (top left). See text for the criteria. Second, IDA also provides genomic data (CNVs and somatic mutations) for ARS/AIMPs and their neighbors in diverse cancer types, which were collected from CanGEM, TumorScape, CCLE and COSMIC (bottom left). Third, IDA contains gene expression data of ARS/AIMPs and their neighbors for 10 cancer types, as well as *P* values and log_2_-fold-changes that represent differential expression of the genes in the 10 cancer types (bottom right). IDA further contains proteomic data (protein abundance and PTMs) collected from dbDEPC, PHOSIDA and PhosPhoSitePlus. Finally, IDA provides PPIs of ARS/AIMPs and their neighbors collected from 15 interactome databases.

Recently, huge amounts of global genomic, transcriptomic and proteomic data for cells or tissues of various cancers have been generated and deposited into numerous databases. Although these data can be useful to understand association of ARS/AIMPs with cancers, different types of global data have been stored in separate databases, which make analyses of ARS/AIMPs inefficient. To resolve this problem, we collected genomic, transcriptomic, proteomic and interaction data of ARS/AIMPs and their interactors from numerous databases and integrated them to IDA. To facilitate analyses of association of ARS/AIMPs with cancers, we first defined cancer-associated genes (CAGs) using NCI cancer gene index (Figure 1, upper left). Among 6955 human genes in the NCI cancer gene index, we selected 3501 CAGs with the following attributes: (i) cancer associations of the genes should be validated by manual curation in the literatures; (ii) the genes should have no conflicting indications regarding their cancer associations; (iii) cancer associations of the genes should be experimentally validated in human samples and (iv) the genes should include one of the following role codes: Chemical_or_Drug_Has_Mechanism_Of_Action, Chemical_or_Drug_Has_Study_Therapeu-tic_Use_For, Chemical_or_Drug_FDA_Approved_for_Disease, Chemical_or_Drug_Has_ Accepted_Therapeutic_Use_For, Gene_Malfunction_Associated_With_Disease and Gene_ Anormaly_has_Disease-Relat-ed_Function.

#### CAGs interacting with ARS/AIMPs

Mammalian ARS/AIMPs have additional domains to catalytic domains (2). They interact with other molecules through the additional domains, thereby affecting activities of cancer-related cellular processes (3). Next, we thus identified the CAGs that interact with ARS/AIMPs among the 3501 CAGs. To this end, we collected 1 12 596 PPIs from the following 15 interactome databases with (Figure 1, upper right): (i) curated PPIs for each of which experimental evidence was previously reported from human protein reference database (24), Kyoto Encyclopedia of Genes and Genomes (25), Biomolecular interaction network database (BIND) (26), Database of Interacting Proteins (27), Biological General Repository for Interaction Datasets (28), Reactome (29), Molecular INTeraction database (30), IntAct (31), InnateDB (32), Search Tool for the Retrieval of Interacting Genes/Proteins (STRING, v9.0) (33) and PharmDB (34) and (ii) predicted PPIs based on paralog- and ortholog-based PPI prediction and text mining from STRING, BIND, interologous interaction database (I2D) (35), online predicted human interaction database (36), human PPI map (HiMAP) (37) and human PPI prediction (38). In addition, the resources that integrate PPIs in individual interactome databases, such as PSICQUIC (EBI) (39) and NCBI PPI databases, were also used. On the basis of the collected PPIs, we identified 123 first neighbor CAGs and 1293 second neighbor CAGs of ARS/AIMPs among the 3501 CAGs.

#### Transcriptomic data of ARS/AIMPs and their interacting CAGs

Alteration of expression levels of the genes in cancers suggests their associations with a broad spectrum of cancer pathophysiological processes. To examine cancer-associated alteration in gene expression, huge amounts of gene expression profiles from diverse types of cancers have been collected (40–43). Thus, we collected 40 cancer expression profiles for 10 representative cancer types (pancreatic, prostate, lung, breast, colon, kidney, head and neck, hematopoietic and lymphoid, liver and gastric cancer) (Figure 1, bottom right). The 164 cancer gene expression datasets were initially collected from the Gene Expression Omnibus (44) and ArrayExpress (45). Among the 164 datasets, 40 were then selected based on the following criteria: (i) each dataset should include more than 10 normal and 10 cancer samples and (ii) each cancer type should include at least two datasets (Supplementary Table S1).

**Figure 2.**
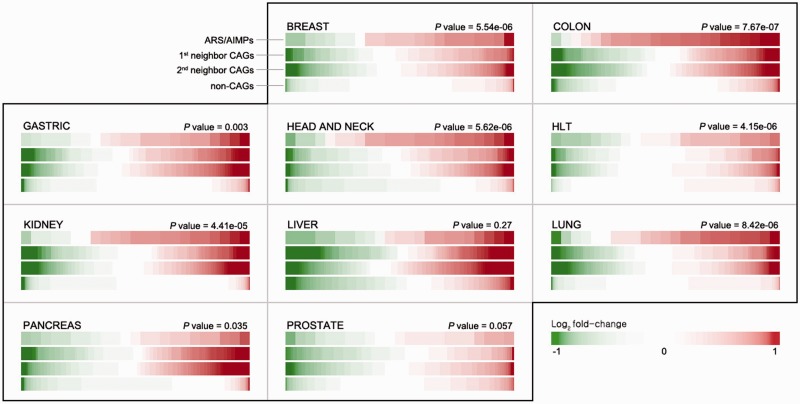
Differential expression of ARS/AIMPs and their neighbors. IDA provides a functionality for exploration of differential expression of the four groups of the genes in the 10 cancer types: (i) ARS/AIMPs, (ii) and (iii) their first and second neighbor CAGs and (iv) non-CAGs. The color bar for each group shows sorted log_2_-fold-changes of the genes in the group. Compared with non-CAGs, the ARS/AIMPs and their interactors showed more significant differential expression in the 10 cancer types. The *t*-test was used to compute *P*-values representing the significances of the differences between the mean log_2_-fold-change values of ARS/AIMPs and non-CAGs. Color bar denotes the gradient of log_2_-fold-changes of the genes. HLT: hematopoietic and lymphoid tissue.

In each dataset, DEGs between cancerous and normal samples were identified using the integrative statistical method previously reported (*P* < 0.05) (17), as described in Materials and Methods. For each cancer type, we then computed representative *P* values of the genes as the combined *P *values of the genes in multiple datasets of the cancer type (19) using Stouffer’s method (Materials and Methods). Also, to summarize expression changes in the 10 types of cancers, the representative log_2_-fold-changes of the genes were computed as their averaged log_2_-fold-changes. For comparative analysis of ARS/AIMPs and their interacting CAGs, we identified 1874 non-CAGs, as negative controls, which were not included in the cancer gene index and also showed no significant cancer-associated alterations in gene expression profiles (i.e. combined *P* < 0.05 in none of the 10 types of cancers). The comparison of the representative log_2_-fold-changes revealed that ARS/AIMPs and their first and second neighbors in the 10 types of cancers showed significant changes in their expression levels, compared with non-CAGs (Figure 2).

#### Genomic data of ARS/AIMPs and their interacting CAGs

Recently, a number of studies have showed that somatic mutations and CNVs were associated with cancer-related pathophysiological processes (40–43, 46). Thus, to understand cancer-related alterations of ARS/AIMPs and their interactors in somatic mutations and CNVs, we first obtained CNV data for nine cancer types (skin, gastric, colon, head and neck, hematopoietic and lymphoid, lung, bone, soft tissue and uterine) from CanGEM (47) and also from Tumorscape (48) for 14 cancer types (leukemia and breast, colorectal, esophageal squamous, lung, liver, ovarian, prostate and renal cancers and medulloblastoma, melanoma, myeloproliferative disorder, glioma and sarcoma) (Figure 1, bottom left; Supplementary Table S2). Frequencies of gain and loss for each gene in individual types of cancers were pre-computed as described previously (47) and stored in IDA. We then obtained somatic mutations from cancer cell line encyclopedia (CCLE) (49) and catalog of somatic mutations in cancer for diverse cancer types (50). IDA contains a total of 7 09 008 somatic mutations including missense, indel, frameshift and nonsense mutations (1452 for ARS/AIMPs, 5 87 125 for their interacting CAGs and 1 20 431 for non-CAGs).

**Figure 3.**
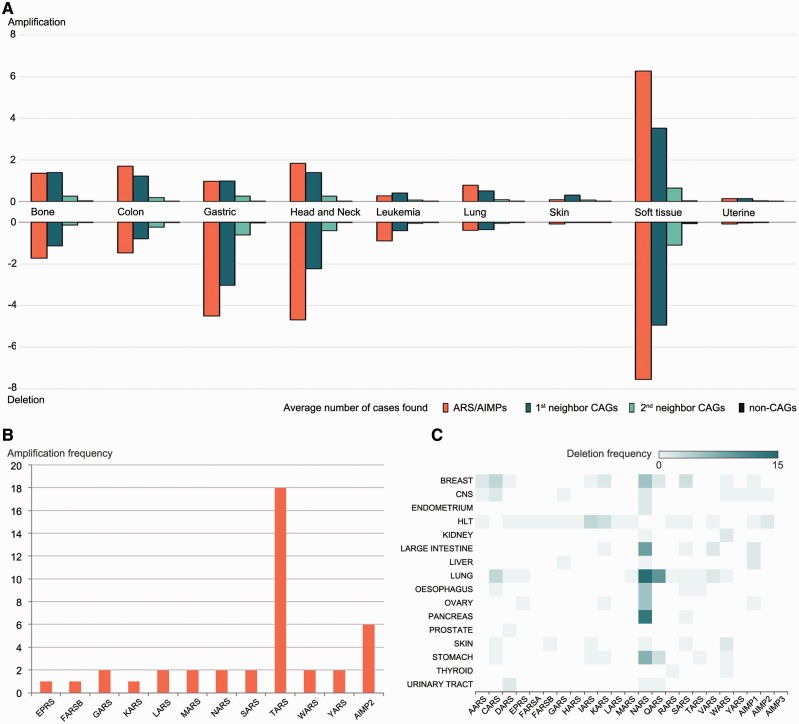
Alterations of copy numbers of ARS/AIMPs and their neighbors. (**A**) Average frequencies of gains (amplification) and losses (deletion) of copy numbers in the four groups of the genes in nine representative cancer types using CNV data in CanGEM: (i) ARS/AIMPs, (ii) and (iii) first and second neighbor CAGs and (iv) non-CAGs. The frequency was defined by the number of amplification cases for the genes in each group divided by the total number of the genes in the group. (**B**) Amplification frequencies of ARS/AIMPs in lung cancer cell lines using CNV data in CCLE. (**C**) Heat map showing deletion frequencies of ARS/AIMPs in 16 different types of cancer cell lines using CNV data in CCLE. Color bar shows the gradient of deletion frequency. CNS, central nervous system; HLT, hematopoietic and lymphoid tissue.

Using these data, we compared alteration degrees of CNVs in ARS/AIMPs, first and second neighbor CAGs and non-CAGs (Figure 3A). ARS/AIMPs and their interactors showed significant gains and losses of copy numbers in nine representative cancers, whereas little variation was observed in non-CAGs. We further examined gains and losses of ARS/AIMPs using the CNV data obtained from CCLE. In total, 18 cases of TARS amplifications were found in lung cancer cells (Figure 3B). High frequencies of NARS deletions were observed in several cancer types, such as large intestine, lung and pancreas cancer cells (Figure 3C). Especially, ∼23% of esophagus cancer cells contained NARS copy number deletions.

**Figure 4.**
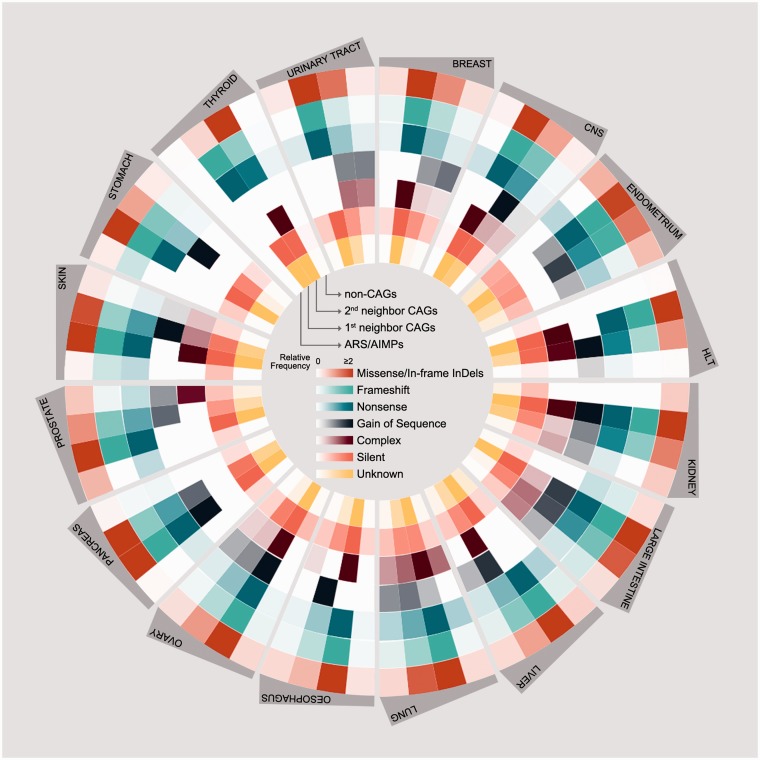
Somatic mutations of ARS/AIMPs and their neighbors. Average numbers of somatic mutations in the four groups of the genes in 16 cancer types using somatic mutation data in CCLE: (i) ARS/AIMPs, (ii) and (iii) first and second neighbor CAGs and (iv) non-CAGs. The mutations were categorized into the following groups: (i) missense/in-frame InDels, (ii) frameshift, (iii) nonsense, (iv) gain of sequence, (v) complex, (vi) silent and (vii) unknown mutations. Color bar denotes the gradient of the number of each group of mutations.

Also, we compared somatic mutations of ARS/AIMPs, first and second neighbor CAGs and non-CAGs (Figure 4). The CAG neighbors showed relatively higher frequencies of somatic mutations (average relative frequency = 5.3) than non-CAGs (average relative frequency = 0.35) in all the cancer types examined. Interestingly, the mutation rates of ARA/AIMPs (average relative frequency = 0.35) were also relatively lower than their CAG neighbors, although ARS/AIMPs showed significant cancer-related alterations in their expression (Figure 2). Nonetheless, several ARS/AIMPs showed relatively high frequency of missense/in-frame indel mutations in several cancer types, such as endometrium and prostate cancers (average relative frequencies = 0.78 and 0.74, respectively). For example, a total of 209 missense/in-frame indel mutations of ARS/AIMPs were found in endometrium cancers. Especially, among the mutations, nearly 80 missense mutations of MARS, CARS, LARS and EPRS were found in the endometrium cancers. Also, the same type of mutations, including both missense and in-frame deletion mutations of KARS, were observed with relative frequency of 0.74 in prostate cancers.

#### Proteomic data of ARS/AIMPs and their interacting CAGs

Post-translation modifications (PTMs) of ARS/AIMPs and their interacting CAGs, such as phosphorylation and ubiquitination, can modulate the interactions of ARS/AIMPs with CAGs, thereby affecting non-canonical functions of ARS/AIMPs in disease-related cellular processes. For example, EPRS, KRS and AIMP2 are dissociated from MSCs by phosphorylation and regulate inflammation, metastasis and apoptosis, respectively (51–53). Thus, we collected PTMs of ARS/AIMPs and their interactors, as well as their protein abundances, in diverse types of cancers (Figure 1, bottom right). First, protein abundance data in 20 cancer types (brain, breast, cervical, colorectal, esophageal, gall bladder, gastric, head and neck, liver, lung, ovarian, pancreatic, prostate, renal, skin, testicular and thyroid cancers and leukemia, lymphoma and sarcoma) were obtained from dbDEPC (54). Second, PTM data of ARS/AIMPs and their interactors were obtained from phosphorylation site database (PHOSIDA) and PhosPhoSitePlus (55, 56). A total of 375 phosphorylations for ARS/AIMPs were found in several cancer cells including cervical, breast and colorectal cancer and leukemia cells. Additionally, IDA includes 84 acetylations, 410 ubiquitinations, 4 methylations and 1 sumoylation for ARS/AIMPs.

### Analytical tools of ARS/AIMPs and their interactors in IDA

#### Web-based search and exploration tools

**Figure 5.**
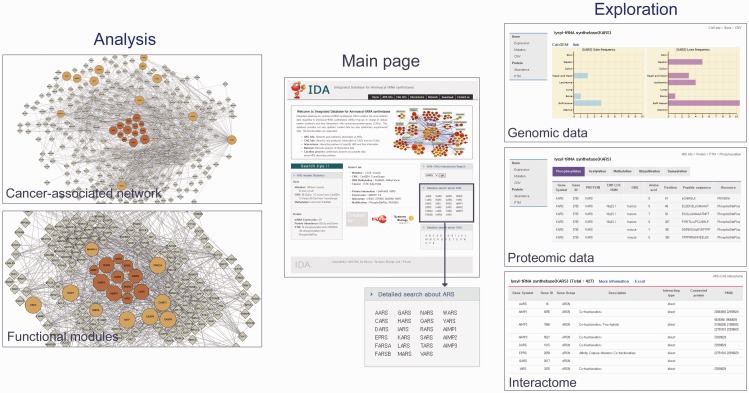
Analytical tools in IDA. IDA provides analytical tools for the search of ARS/AIMPs and their interactors (right), reconstruction of ARS/AIMP network models (top left) and identification of key functional modules in the network (bottom left). These example networks in the left panel were generated using ‘Cancer-associated network’ (top) and ‘Functional modules’ (bottom), respectively, in ‘Analysis’ of ‘Network’ menu. KARS was entered as an input, and a network (top left) was generated by clicking ‘Submit’ button after selecting first and second neighbor CAGs. The main page shows the interface for the search of ARS/AIMPs and their interacting CAGs (center). The exploration panels (right) show the search outputs: (i) genomic data of KARS (gains and losses of CNVs), (ii) proteomic data of KARS (PTMs) and (iii) PPIs of KARS. Detailed information for PTMs and CAG interactors of ARS/AIMPs can be found through the links to the original databases. The network modeling and analysis tools (left) show the ARS/AIMP network model and key functional modules identified from the network model. Colored nodes represent ARS/AIMPs (dark orange) and their first neighbors (orange). Node sizes represent the average deregulation scores in the 10 cancer types (see Materials and Methods).

The goal of IDA is to provide not only molecular and interaction data of ARS/AIMPs but also analytical tools that generate network models and then network-driven hypotheses for understanding of cancer-associated functions of ARS/AIMPs. IDA provides a search tool (‘Detailed search about ARS’ in Figure 5, center) that allows us to explore the collected genomic, transcriptomic and proteomic data for ARS/AIMPs. For example, when the genomic data for DARS were searched, the search output shows the genomic, proteomic and interaction data of DARS in IDA. The genomic data showed the gains of copy numbers in soft tissue cancers, but losses of copy numbers in gastric, head and neck and soft tissue cancers (Figure 5, top right). The proteomic data showed the five types of PTMs for DARS (phosphorylation, acetylation, methylation, ubiquitination and sumoylation), as well as the PTM sites (Figure 5, middle right): For example, the phosphorylation data showed five serine (S112, S146, S238, S249 and S427), three tyrosine (Y24, Y239 and Y245) and one threonine (T52) phosphorylation sites. Further detailed information of the PTMs can be obtained through the links to the original PTM databases.

Moreover, another search tool (‘ARS-CAG interactome search’ in Figure 5, center) allows us to explore the CAGs with which ARS/AIMPs interact. For example, the search output for KARS showed a list of CAGs interacting with KARS (Figure 5, bottom right). This list of interactors of KARS can be used to study their KRS-related functions and pathological implications such as cell migration (MAPK11/12/14) (52, 57), immune response (MITF) (57) and Amyotrophic Lateral Sclerosis (SOD1) (58, 59), supporting the utility of the interactors of ARS/AIMPs. Finally, the first and second CAG neighbors of ARS/AIMPs can be explored using the links to the list of interacting CAGs or the search tool for the CAGs (‘Detailed search about CAG’ in Figure 5, center). All the genomic and proteomic data of the CAGs can be explored, similar to those of ARS/AIMPs shown in Figure 5. Finally, the interactome of a list of genes with the molecules in IDA can be obtained using ‘Cancer-associated network’ in ‘Analysis’ of the ‘Network’ menu after entering the symbols of the genes (‘Select All’ group option).

#### Cancer-associated network models for ARS/AIMPs

**Figure 6.**
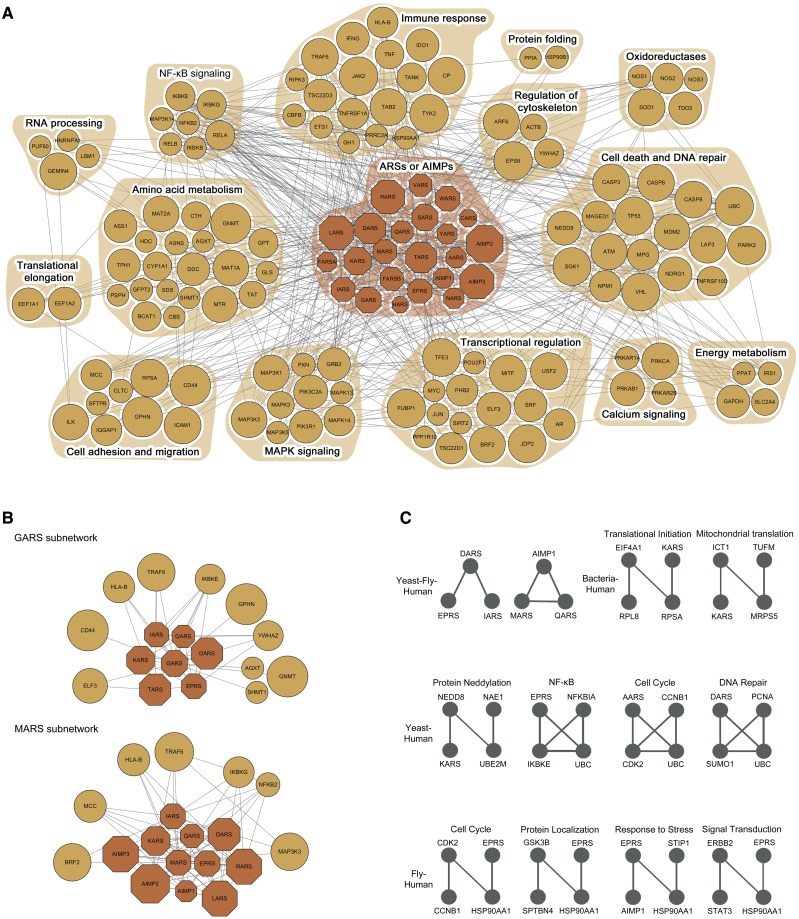
Evolutionarily conserved motifs in the ARS/AIMP network model. (**A**) ARS/AIMPs cancer network model showing 23 ARS/AIMPs and 123 first neighbor CAGs. Colored nodes represent ARS/AIMPs (dark orange) and their first neighbors (orange). Node sizes represent the average deregulation scores in the 10 cancer types (Materials and Methods). Nodes were grouped, such that the nodes involved in the same cellular process, according to gene ontology biological process annotations, belonged to a functional module denoted by the orange background. (**B**) Two key functional modules identified using the random walk-based method. (**C**) Evolutionarily conserved motifs in the ARS/AIMP network models constructed in human, fly, bacteria and yeast. The motifs conserved between human and the other three species were shown.

An important goal of the tools in IDA is to develop cancer-associated network models for ARS/AIMPs by integrating both transcriptomic and interaction data and to generate hypotheses for mechanisms of ARS/AIMPs in cancer-related pathophysiological processes based on the network models. To this end, IDA first provides the tools to develop cancer-associated network models for ARS/AIMPs (‘Cancer-associated network’ in ‘Analysis’ of ‘Network’ menu). The network model was generated using ARS/AIMPs and their first neighbor CAGs and then visualized using Cytoscape (60) (Figure 5, top left). The nodes (ARS/AIMPs and their first neighbor CAGs) in the network model can be organized into functional modules in each of which the nodes have the same gene ontology biological processes (Figure 6A; ‘Cancer-associated network of ARS/AIMPs and first neighbor CAGs’ in ‘Output’ of ‘Network’ menu). The nodes in the network model can have different degrees of contributions to cancer-related pathophysiological processes. To represent the different degrees of node contributions to the cancer-related processes, we estimated deregulation score indicating representative degree of deregulation in the 10 cancer types for which gene expression data were collected (Materials and Methods). The interactions (edges) between ARS/AIMPs and CAGs in the network model can have different degrees of associations with cancers. To represent the different degree of cancer associations for each pair of ARS/AIMP and its CAG interactor, we estimated the co-association score for the interacting pair that represents the extent of co-deregulation in the 10 cancer types. The network model can reveal a set of the linked nodes with high deregulation and co-association scores, which can be considered as a functional module that can significantly contribute to cancer-related pathophysiological processes represented in the network model.

#### Key functional modules of ARS/AIMPs in the network model

To understand functions of ARS/AIMPs defined by their interactions with the CAGs, it is important to identify the sets of the ARS/AIMP-CAG nodes densely connected with high deregulation and co-association scores, which are referred to as key functional modules of ARS/AIMPs. These modules should include at least one ARS/AIMP and its interaction with the CAGs to examine cancer-related functions of ARS/AIMPs. Thus, IDA provides a random walk-based method to effectively identify the key ARS/AIMP-CAG functional modules (‘Functional modules’ in ‘Analysis’ of ‘Network’ menu). This method performs random walks based on the co-association scores and then identifies a set of the nodes that should include more than at least one ARS/AIMP with high residence probabilities after a certain period of time. For the network model in Figure 6A, the random walk-based method generated two key functional ARS/AIMP-CAG modules (Figure 6B; ‘Functional modules for cancer-associated network of ARS/AIMPs and first neighbor CAGs’ in ‘Output’ of ‘Network’ menu). These modules (GARS and MARS subnetworks) show the links of the ARSs to (i) NFKB pathway through their interactions with NFKB2, IKBKG/E and MCC (61, 62) and (ii) transcriptional regulators (ELF3 and BRF2). This suggests potential roles of GARS and MARS in NFKB signaling and transcriptional regulation. Furthermore, these modules show the possible association of the ARSs in the immune response (HLA-B and TRAF6) and cell migration (CD44 and MCC).

#### Evolutionary analysis of the cancer-associated network for ARS/AIMPs

In addition to the random walk-based method mentioned above, evolutionary analysis of the cancer-associated network for ARS/AIMP can be applied to the network model to identify core regulatory motifs of ARS/AIMPs, assuming that the core regulatory motifs should be conserved across different species. To this end, we collected PPIs in four different species, *Escherichia coli, Saccharomyces cerevisiae, Drosophila melanogaster* and *Homo sapiens* (Supplementary Table S3) and then reconstructed ARS/AIMP network models using first and second neighbors of ARS/AIMPs in individual species. Next, we identified evolutionarily conserved network motifs recurring in the network models of individual species (‘Evolutionarily conserved motifs’ in ‘Analysis’ of ‘Network’ menu). Among the identified motifs, we focused on the ones conserved in human to understand roles of ARSs in human networks (‘Evolutionarily conserved motifs of ARS/AIMPs and first and second neighbor CAGs’ in ‘Output’ of ‘Network’ menu). The identified motifs were mostly conserved in two species, Yeast-Human (4 motifs), Fly-Human (4 motifs) and Bacteria-Human (2 motifs) (Figure 6C).

These evolutionarily conserved motifs suggest that the components in the motifs may form functional complexes. For instance, the motif consisting of MARS, AIMP1 and QARS was suggested to be conserved among yeast, fly and human. These three proteins are the known components forming MSC with other ARSs in human (63). In the human form, the structural or functional association AIMP1 with QARS and MARS were experimentally demonstrated (4, 64). The primitive complex form was found in yeast consisting of MARS, Acr1p (homolog of AIMP1) and EARS (65) and the functional significance of this complex was studied in-depth (66). The potential interactions among these three proteins were also suggested in fly (67), indicating that functional implications of the fly complex can be obtained from the in-depth studies conducted in other organisms. Thus, the conserved motif analysis can be useful to predict how these ARS-containing macromolecular complexes have evolved from unicellular to multicellular organisms in their structure and function.

The nodes in the conserved motifs are involved in a broad spectrum of cellular processes, including cell cycle and DNA repair, protein modification (e.g. neddylation) and localization, signal transduction and response to stress (e.g. NFKB pathway). These results indicate that ARSs can play potential roles in these processes. Interestingly, the link of ARS/AIMPs to NFKB pathway was predicted by both the random walk-based method and the evolutionary analysis of ARS/AIMP networks. Many motifs conserved in higher organisms, as well as a broad spectrum of cellular processes associated with these motifs, are consistent to previous findings that they can acquire evolutionarily new functions by the addition of new domains (68–70).

## Conclusions

Several studies have discovered diverse implications of ARS/AIMPs in human diseases. However, there is still the lack of data resources and tools that can be used to analyze potential functional roles of ARS/AIMPs in human diseases. IDA was developed to provide a broad spectrum of genomic (somatic mutations and CNVs), transcriptomic (mRNA expression), proteomic (protein abundance and PTMs) and interactome data (PPIs) from 25 existing DBs (Figure 1) for ARS/AIMPs and their interactors. However, IDA is not simply assemblies of information available in the 25 DBs that provide intact or federated links to the original data. The collected data were reprocessed, designed and integrated in such a way to facilitate the integrative analyses (analyses of cancer-related changes in gene and protein abundances and genomic variations, network analysis, functional modules and evolutionary motif analysis) and explorative analyses (navigation and visualization of the information) of cancer-related ARS data. When cancer-related alterations in abundances and genomic variations are discovered for ARS/AIMPs and/or their interactors, IDA can be used to evaluate whether the discovery was further investigated or not. Furthermore, IDA provides a battery of analytical tools for the integrative analyses and also the outputs from the integrative analyses. The outputs (e.g. network modules) can be used to generate hypotheses for functions of ARS/AIMPs or mechanisms for such functions in cancers, thereby providing new insights into cancer-related functions of ARS/AIMPs. Currently, IDA provides cancer-related data only and is thus limited to investigation of association of ARS/AIMPs with cancers. However, it will be expanded to include genomic, transcriptomic and proteomic data collected from metabolic and neurological diseases. Recently, a number of studies have shown important roles of ARS/AIMPs in pathogenesis of diverse diseases. Therefore, IDA can serve as a comprehensive resource of data and tools for studies of ARS/AIMPs that can facilitate elucidation of unknown functions of ARS/AIMPs in disease pathogenesis.

## Funding

This work was supported by Global Frontier Project funded by the Ministry of Science, ICT and Future Planning and Basic Science Research Program funded by the Ministry of Education (NRF-M3A6A4-2010-0029785 and NRF-2013R1A1A2058353) and the grants from the Institute for Basic Science (CA1308), Next-Generation BioGreen 21 Program (No. PJ009072) and the POSCO Research Fund (Project No. 2013Y008). Funding for open access charge: Global Frontier Project (NRF-M3A6A4-2010-0029785).

## Supplementary Data

Supplementary data are available at *Database* Online.

*Conflict of interest.* None declared.
